# A CDK activity buffer ensures mitotic completion

**DOI:** 10.1242/jcs.259626

**Published:** 2022-06-21

**Authors:** Souradeep Basu, James O. Patterson, Theresa U. Zeisner, Paul Nurse

**Affiliations:** 1 Cell Cycle Laboratory, The Francis Crick Institute, 1 Midland Road, London NW1 1AT, UK; 2 Laboratory of Yeast Genetics and Cell Biology, Rockefeller University, 1230 York Avenue, New York, NY 10065, USA

**Keywords:** Cyclin-dependent kinase, CDK inhibitor, Cdk1, Mitosis, Kinase activity, *Schizosaccharomyces pombe*

## Abstract

The eukaryotic cell cycle is driven by the activity of cyclin-dependent kinases (CDKs). CDK activity rises over 50-fold during the cell cycle, from a low level in G1 to a high level in mitosis. However, it is not known whether the entire range of CDK activity is necessary for cell cycle progression, or whether cells can tolerate a reduction in CDK activity level. Here, in fission yeast, we show that sublethal CDK inhibition lengthens the time cells spend in mitosis but does not cause misordering of mitotic events. Maximum attainable CDK activity exceeds the amount necessary for mitosis, and thus forms a CDK activity buffer between sufficient and maximal possible CDK activities. This CDK activity buffer is needed for mitotic completion when CDK activity is compromised, and CDK inhibition only becomes lethal to cells when this buffer is exhausted. Finally, we explore what factors influence this CDK activity buffer, and find that it is influenced by CDK-counteracting phosphatases. Therefore, maximum attainable CDK activity is not necessary for mitosis but provides robustness to CDK activity reduction to ensure mitotic completion.

## Abstract (precis)

**Summary:** CDK activity drives the eukaryotic cell cycle. We show that CDK activity is in surplus, and cells use this surplus to ensure mitotic completion despite CDK inhibition.

## INTRODUCTION

The major cell cycle events of DNA replication and mitosis are driven and ordered by the activity of cyclin-dependent kinases (CDKs) (Hayles et al., 1994; Kõivomägi et al., 2011; Swaffer et al., 2016). Low CDK activity at the G1-S transition is responsible for DNA replication, but higher CDK activity at mitosis is responsible for phosphorylating hundreds of CDK substrates that are necessary to coordinate the complex cellular reorganisation needed for chromosome separation and cell division (Holt et al., 2009; Pagliuca et al., 2011; Zegerman and Diffley, 2007). CDK activity rises in a burst-like manner at mitotic entry, which provides a CDK activity increase of up to 50-fold between G1 and mitosis (Pomerening et al., 2005, 2003; Swaffer et al., 2016). Although it is known that CDK activity rises to a high level at mitosis, it is unknown whether this high level of activity, and thus the broad dynamic range of CDK activity, is actually necessary for cell cycle progression *in vivo*. Therefore, we asked whether cells were able to tolerate a lower level of CDK activity than the maximum attainable CDK activity and still be capable of undergoing mitosis.

## RESULTS AND DISCUSSION

### Cells are resilient to CDK inhibition

**Fig. 1.**
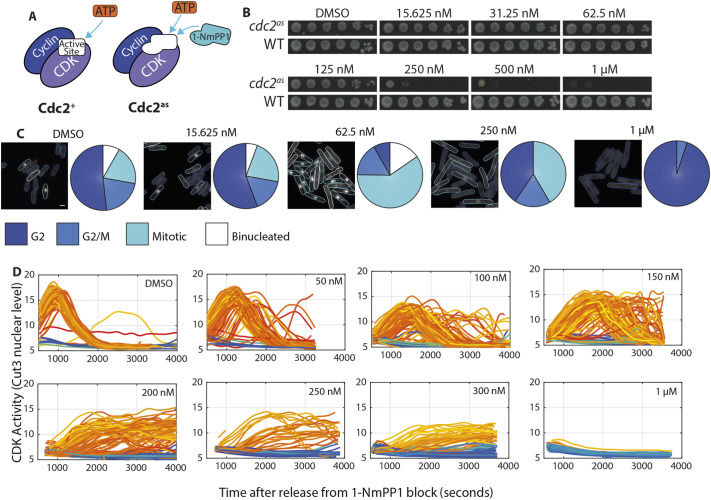
**Cells are resilient to CDK inhibition until a threshold concentration of inhibitor.** (A) Schematic of cyclin–CDK complexes formed by either wild-type Cdc2 (Cdc2^+^) or Cdc2(as) (Cdc2^as^) with cyclin. Cdc2(as) possesses F84G and K79E mutations that allow 1-NmPP1 to inhibit Cdc2. (B) Serial dilution assay of wild-type cells (WT) and cells carrying *cdc2(as)* (Cdc2^as^) in the presence of the indicated 1-NmPP1 concentrations. DMSO was used as a vehicle control. Cells were grown on EMM4S for 4 days at 32°C. Data shown are representative of four biological repeats. (C) Representative images of Cdc2^as^ cells expressing Cut3–GFP after treatment with the annotated concentrations of 1-NmPP1. Cell cycle stages were assigned based on Cut3–GFP nuclear:cytoplasmic intensity ratio (see Materials and Methods). Pie charts show cell cycle stage proportions within the population. Data shown are representative of three biological repeats. Scale bar: 5 μm. (D) Single-cell CDK activity traces versus time for Cdc2^as^ cells in the presence of the indicated 1-NmPP1 concentrations. CDK activity is represented by the nuclear intensity level of Cut3–GFP. Cells were automatically assigned as mitotic or non-mitotic (see Materials and Methods). Orange traces indicate mitotic cells. Blue traces indicate non-mitotic cells. DMSO, *n*=73 single-cell traces; 50 nM, *n*=85 cells; 100 nM, *n*=137 cells; 150 nM, *n*=110 cells; 200 nM, *n*=100 cells; 250 nM, *n*=41 cells; 300 nM, *n*=88 cells; 1 μM, *n*=52 cells. Data shown are aggregated from two biological repeats.

To probe whether maximal CDK activity is needed physiologically for mitosis, we progressively reduced *in vivo* CDK activity using a fission yeast strain carrying an analogue-sensitive (as) variant of the gene encoding the only cell cycle CDK in fission yeast, *cdc2* (Fig. 1A). This *cdc2-asM17* [referred to hereafter as *cdc2(as)*]-carrying strain is sensitive to inhibition by the ATP analogue 1-NmPP1 (Aoi et al., 2014).

Assaying cell viability in increasing concentrations of 1-NmPP1 showed that whilst wild-type cells were able to withstand 1 μM 1-NmPP1 treatment, *cdc2(as)* cells lost viability at 1-NmPP1 concentrations above 250 nM (Fig. 1B). The *cdc2(as)* cells remained viable at 1-NmPP1 concentrations up to 125 nM, suggesting that cells may not require maximum levels of CDK activity for mitotic progression. We then examined the cell cycle profiles of 1-NmPP1-treated cells by using Cut3–GFP as a marker of CDK activity. Cut3 translocates into the nucleus at mitosis, and its nuclear levels serve as an *in vivo* readout of CDK activity (Patterson et al., 2021). This can be used to assign cells as being mitotic, at the transition of G2-M or in interphase (see Materials and Methods). This analysis revealed that at sublethal 1-NmPP1 doses, more cells were present at the transition into mitosis and in mitosis itself (Fig. 1C), suggesting that the time to progress through mitosis was extended because it was occurring at a slower rate.

We confirmed this observation through time-lapse microscopy using Cut3–GFP as a marker of CDK activity. After release from a G2 block we observed that without any CDK inhibition, CDK activity rose and fell rapidly (Fig. 1D, DMSO panel). However, with CDK inhibition, cells spent progressively longer in mitosis (Fig. 1D). Cells also spent a longer time entering mitosis after release, as the halting of cell elongation (a marker of early mitosis) was progressively delayed in increasing concentrations of 1-NmPP1 (Fig. S1). We conclude that sublethal 1-NmPP1 levels cause a slower progression into and through mitosis, and at lethal levels completely blocks the onset of mitosis.

**Fig. 2.**
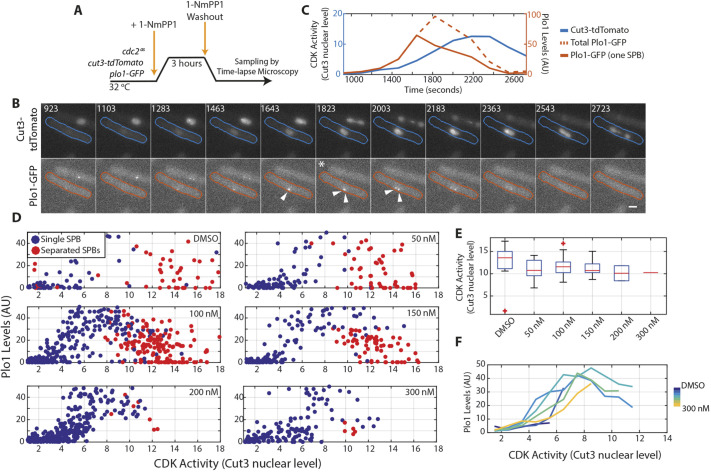
**Robust mitotic ordering is maintained with inhibited CDK.** (A) Experimental outline for the data shown in B–F. Cells carrying *cdc2(as)* (*cdc2^as^*) and expressing Cut3–tdTomato and Plo1–GFP were blocked with 1 μM 1-NmPP1 for 3 h before being released into various concentrations of 1-NmPP1 (washout). (B) A representative cell from the experiment described in A following release from 1-NmPP1 into DMSO. Upper row: Cut3–tdTomato. Lower row: Plo1–GFP. Time elapsed after release is shown in seconds, and the first time point after SPB separation is marked with an asterisk. Arrowheads indicate SPBs at and around this time point. Cell outlines highlight the cell quantified in C. Scale bar: 2 μm. (C) Quantification of CDK activity (assayed using Cut3–tdTomato) and Plo1–GFP fluorescence intensity levels for the cell indicated in B. (D) Scatter plot of Plo1–GFP levels versus CDK activity (as measured using the nuclear intensity level of Cut3–tdTomato) after release into the indicated 1-NmPP1 concentrations or DMSO as a vehicle control. DMSO, *n*=105 measurements; 50 nM, *n*=146 measurements; 100 nM, *n*=446 measurements; 150 nM, *n*=189 measurements; 200 nM, *n*=312 measurements; 300 nM, *n*=149 measurements. Data shown are representative of two biological repeats. (E) Boxplots of CDK activity level at the first instance of SPB separation for cells released into the indicated 1-NmPP1 concentrations. Box delimits the 25th and 75th percentiles. Whiskers delimit the 10th and 90th percentiles. Middle line indicates the median. Whiskers are not shown for *n*≤10, box and whiskers are not shown for *n*≤5. Data are taken from panel D. (F) Binned means of Plo1–GFP levels against CDK activity at the indicated 1-NmPP1 concentrations. Bin windows: 0.5 AU. Data are taken from panel D. AU, arbitrary units.

It has previously been suggested that mitotic events occur at defined CDK activity thresholds (Deibler and Kirschner, 2010; Gavet and Pines, 2010), but the lack of CDK inhibition lethality at lower inhibitor concentration argues that mitotic ordering must be robust to changes in CDK activity. To test this, we used two markers of mitotic progression – spindle pole body (SPB) separation and Polo kinase activation – and assayed whether they were consistently triggered at a defined level of CDK activity, or whether inhibition of CDK caused misordering of these mitotic events. We used Cut3–tdTomato levels as a readout for CDK activity, monitored Polo activation by the level of GFP-tagged Plo1 kinase at the SPB, and also followed the timing of SPB separation (Fig. 2A–C) (Basu et al., 2020; Kamenz et al., 2015). The CDK activity at which SPB separation occurred was slightly higher in DMSO-treated cells compared with that in 1-NmPP1-treated cells, but was consistent from 50 nM to 300 nM of 1-NmPP1, indicating that SPB separation occurs at a defined level of CDK activity (Fig. 2D,E). Plo1 levels were also reasonably overlapping when plotted against CDK activity (Fig. 2D,F). Therefore, when CDK activity is inhibited, molecular events are still dependent on, and ordered by, cells attaining CDK activity thresholds.

### A CDK activity buffer allows robustness to CDK inhibition

The results reported above suggest that cellular CDK activity can rise to above what is actually needed to progress properly through mitosis. To probe the levels of maximum CDK activity during mitosis, we assayed *in vivo* CDK activity in cells that naturally proceeded through mitosis and compared it to that in cells blocked in mitosis, after the addition of increasing amounts of CDK inhibitor. To carry out this experiment, we blocked cells in G2 and released them into either a normal mitosis or a metaphase block by depleting the mitotic APC/C targeting subunit Cdc20 (encoded by *slp1* in fission yeast) using a thiamine-repressible promoter (Petrova et al., 2013). This allowed comparison between CDK activity achieved in a normal cell cycle and the maximum attainable steady-state CDK activity in a mitotic block.

**Fig. 3.**
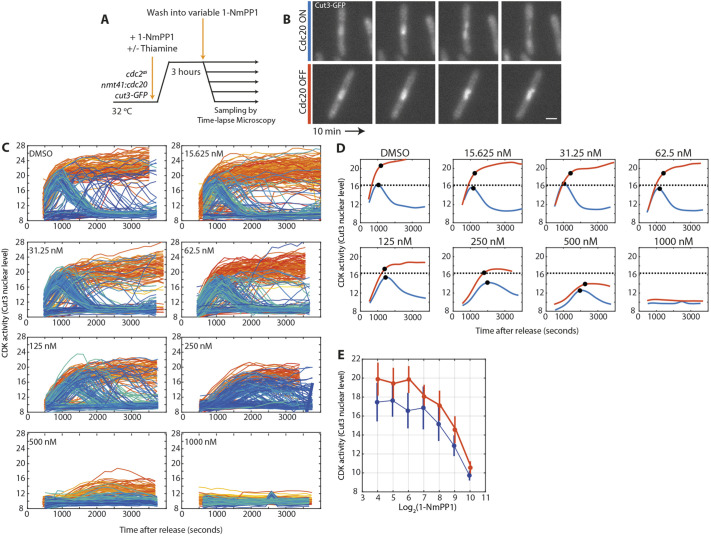
**A CDK activity buffer underlies cellular robustness to CDK inhibition.** (A) Experimental outline for the data shown in B–E. (B) Representative images of Cut3–GFP fluorescence in cells after release from G2 block. Upper row: no thiamine, Cdc20-ON cells. Lower row: thiamine added, Cdc20-OFF cells. Time-lapse images taken every 10 min. Scale bar: 5 μm. (C) Single-cell traces of Cut3–GFP nuclear intensity level after release from G2 arrest into the indicated 1-NmPP1 concentrations. Orange lines mark Cdc20-OFF cells. Blue lines mark Cdc20-ON cells. DMSO, *n*=181 single-cell traces; 15.625 nM, *n*=228 cells; 31.25 nM, *n*=195 cells; 62.5 nM, *n*=240 cells; 125 nM, *n*=185 cells; 250 nM, *n*=211 cells; 500 nM, *n*=193 cells; 1000 nM, *n*=160 cells. Data shown are representative of two biological repeats. (D) Mean of single-cell traces from panel C. Red line, Cdc20-OFF; blue line, Cdc20-ON. For the red trace, the black data point marks the point of maximum rate deceleration; for the blue trace, the black data point marks the peak CDK activity level. Black dashed line represents peak CDK activity in the DMSO release for Cdc20-ON cells, transposed to all other panels. (E) Maximum rate deceleration points (red) or peak CDK activity points (blue) at different 1-NmPP1 concentrations, presented as mean±s.d. Data are taken from panel C.

We released cells into mitosis in the presence or absence of Cdc20, and observed that almost all Cdc20-depleted (Cdc20-OFF) cells became arrested, with high levels of nuclear Cut3, indicating high CDK activity (Fig. 3A,B). In contrast, cells without Cdc20 depletion (Cdc20-ON) first increased their CDK activity before decreasing their activity and exiting mitosis (Fig. 3B). This experiment was repeated with varying concentrations of 1-NmPP1. The maximum CDK activity achieved decreased as 1-NmPP1 concentration increased in both Cdc20-ON and Cdc20-OFF cells (Fig. 3C). Averaging these single-cell traces revealed that, in the absence of CDK inhibition, the maximum attainable CDK activity in Cdc20-OFF cells was ∼30% higher than needed for mitosis (Fig. 3D,E). However, upon increasing CDK inhibition, the maximum attainable CDK activity progressively reduced in Cdc20-OFF cells until, at 250 nM 1-NmPP1, the maximum activity fell to a level similar to the amount needed to complete a normal mitosis in Cdc20-ON cells. This value coincides with the level of 1-NmPP1 at which unperturbed cells rapidly lost viability (Fig. 1B). Above 250 nM 1-NmPP1, peak CDK activity decreased in both Cdc20-ON and Cdc20–OFF cells and remained below the threshold value needed for mitotic completion (Fig. 3D,E).

Therefore, the maximum attainable CDK activity is above that needed for mitotic completion in a normal cell cycle. Thus, there is a CDK activity buffer between the CDK activity level that is sufficient and the maximum level that is achievable. CDK inhibition first consumes this buffer of CDK activity before becoming lethal to cells. This explains why in an unperturbed mitosis (Cdc20-ON state) addition of 1-NmPP1 up to 125 nM did not affect the CDK activity reached, as cells only needed to reach the mitotic threshold of CDK activity, which was still below the maximum attainable activity. However, when this activity buffer is depleted, CDK inhibition is sufficient to block mitosis, leading to lethality. Thus, during a normal mitosis, cells do not reach the maximum attainable level of CDK activity. Instead, cells trigger anaphase and cyclin destruction before the maximum CDK activity is reached.

### Removal of the Cdc14-type phosphatase Clp1 extends the CDK activity buffer

We hypothesised that increasing overall CDK activity would increase this CDK activity buffer and make cells more resistant to CDK inhibition. In eukaryotes, CDK activity is restrained by the activity of Wee1 kinase, which phosphorylates CDK residues Y15 and T14 (Berry and Gould, 1996; Russell and Nurse, 1987). The T14A Y15F (AF) CDK mutant bypasses this regulation, but in fission yeast, this is lethal unless the B-type cyclin Cdc13 is fused to Cdc2 (Coudreuse and Nurse, 2010). However, introduction of the AF mutations into a Cdc13–Cdc2(as) fusion protein did not increase the CDK activity buffer (Fig. S2A).

**Fig. 4.**
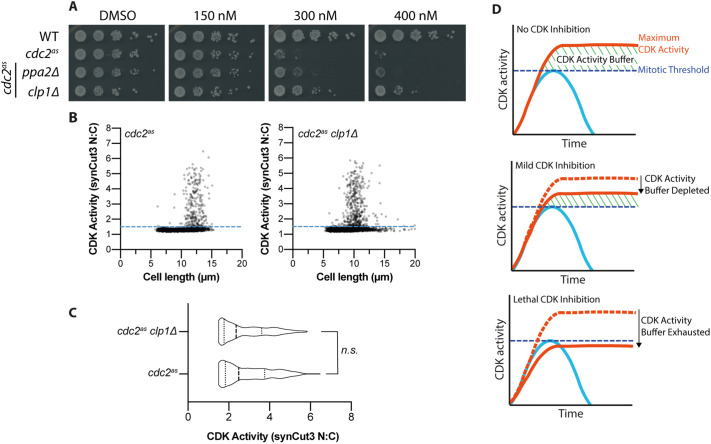
**Certain CDK-counteracting phosphatases influence the CDK activity buffer.** (A) Serial dilution assay of wild-type cells (WT), *cdc2(as)* cells (*cdc2^as^*), and *cdc2(as)* cells with *ppa2*Δ or *clp1*Δ. The concentration of 1-NmPP1 is shown. DMSO was used as a vehicle control. Cells were grown on YE4S for 4 days at 25°C. Data shown are representative of two biological repeats. (B) Scatter plot of CDK activity versus cell length for asynchronous *cdc2(as)* and *cdc2(as) clp1*Δ cells expressing synCut3–mCherry. CDK activity was calculated as the nuclear:cytoplasmic ratio of synCut3–mCherry fluorescence. *n*=6007 cells for *cdc2(as)*; *n*=5734 for *cdc2(as) clp1*Δ. Blue dashed line indicates the mitotic threshold of CDK activity (>1.5). Data shown are representative of two biological repeats. (C) Violin plot of cells with mitotic CDK activity (>1.5 in panel B). Probability that *cdc2(as)* and *cdc2(as) clp1*Δ form part of the same distribution, *P*=0.231 (n.s., not significant; Mann–Whitney rank comparison test). *n*=282 cells for *cdc2(as)*; *n*=461 cells for *cdc2(as) clp1*Δ. Middle dashed line marks the median. Left and right dotted lines mark the 25th and 75th quartile values, respectively. (D) Summary schematic of the CDK activity buffer.

We next expanded our search for activity-buffer determinants to CDK-opposing protein phosphatases. In fission yeast, CDK is opposed by two major protein phosphatases: protein phosphatase 2A (PP2A) and the Cdc14-type phosphatase Clp1 (Kinoshita et al., 1990; Trautmann et al., 2001). Therefore, we deleted the gene encoding the major catalytic subunit of PP2A (*ppa2*) and the gene encoding Clp1 (*clp1*) in two different strains in a *cdc2(as)* background (Fig. 4A).

PP2A is thought to be the major CDK-opposing phosphatase (Mochida et al., 2009) but, in this assay, deletion of *ppa2* had less effect on the level of tolerable CDK inhibition compared with deletion of *clp1* (Fig. 4A). Removal of Clp1 resulted in rescue of 1-NmPP1-mediated lethality at a concentration of 400 nM, showing that cells had become substantially more resistant to CDK inhibition and suggesting that the CDK activity buffer had been extended (Fig. 4A). That deletion of *ppa2* was less effective in extending the buffer compared to *clp1* was surprising, and therefore we considered that the *ppa1* gene product, which encodes a second PP2A catalytic subunit, may also provide PP2A activity. Deletion of *ppa1* resulted in similar levels of viability increase to the deletion of *ppa2* (Fig. S2B), but we were unable to test whether these effects were additive because the *ppa1*Δ*ppa2*Δ strain is inviable (Kinoshita et al., 1990). We also considered that different PP2A targeting subunits may influence the CDK activity buffer in separate ways. However, deletion of the genes encoding the B55 (*pab1*) or the two B56 (*par1* and *par2*) regulatory subunits did not result in increased cell viability at higher concentrations of 1-NmPP1 (Fig. S2B).

Given that removal of Clp1 resulted in the greatest increase in the buffer region, we investigated the effects of Clp1 deletion further to determine how the CDK activity buffer had been extended. Deletion of Clp1 could increase maximum overall CDK activity, or alternatively could extend the buffer region by reducing the CDK activity threshold required for mitosis. We tested this by analysing whether peak CDK activity increased in mitotic cells, using Cut3 as a CDK activity readout (Fig. 4B). We observed that *clp1*Δ cells achieved similar maximum levels of nuclear Cut3 to wild-type cells, suggesting that overall CDK activity was not increased (Fig. 4C). Therefore, we suggest that deletion of Clp1 extends the CDK activity buffer by reducing the CDK activity threshold necessary for mitosis.

Here, we have shown in fission yeast that cells are generally resistant to CDK inhibition due to the presence of a ‘buffer’ of excess CDK activity. At a threshold level of CDK inhibition, this residual activity buffer becomes fully depleted, and mitosis cannot take place (Fig. 4D). Thus, the maximum attainable CDK activity exceeds the activity necessary for mitosis, ensuring that mitotic completion is still possible when CDK activity is partially reduced. The buffer itself can be considered as the difference between the maximum attainable CDK activity and the CDK activity needed to net phosphorylate the final substrate essential for mitosis. Although Cut3 was used as a CDK activity reporter here, CDK substrate sensitivity to CDK activity varies over orders of magnitude (Swaffer et al., 2016), and it is unlikely that Cut3 phosphorylation exactly reflects that of the final substrate essential for mitosis.

Why would cells require a CDK activity buffer? One possibility is resilience against molecular noise and, thus, the variability in CDK activity in different cells. Another reason might be that in some circumstances a cell may need to divide with lower levels of cyclin–CDK complex than usual. Since the levels of cyclin–CDK complex scale with cell size (Patterson et al., 2021; Patterson et al., 2019), this phenomenon might apply to situations where small cells undergo division, such as in conditions of limiting nutrients or where cells are under stress. In these situations, cells would be able to utilise the ‘buffer’ of CDK activity to complete mitosis even though CDK activity is reduced. It is also possible that the CDK activity buffer plays a role in the regulation of CDK behaviour and progression through the cell cycle in physiological conditions.

The molecular mechanisms behind the buffer are unclear, and while unlikely to arise from CDK inhibitory phosphorylation (Fig. S2A), the buffer might relate to cell-size-related factors, such as the accumulation of cyclin–CDK complex over the cell cycle, or CDK activity regulators such as DNA concentration (Patterson et al., 2021). However, investigation of the influence of phosphatase activity on the buffer revealed that deletion of the Cdc14-type phosphatase Clp1 increases the tolerance of cells to CDK inhibition more than the deletion of either of the genes (*ppa1* and *ppa2*) encoding the PP2A catalytic subunit. However, although removal of Clp1 has the greatest effect on the CDK activity buffer on its own, compensation between the two redundant PP2A catalytic subunits may mask the true effect of PP2A activity on the CDK activity buffer. Given that deletion of Clp1 has no effect on the phosphorylation of the model reporter substrate we used, this influence on the CDK activity buffer is likely to work through reducing the CDK activity threshold needed for the final substrate essential for mitosis.

## MATERIALS AND METHODS

### *Schizosaccharomyces pombe* genetics and cell culture

*S. pombe* media and standard methods are as previously described (Moreno et al., 1991). Experiments were conducted in EMM4S medium (Edinburgh Minimal Medium, supplemented with adenine, leucine, histidine and uracil to a final concentration of 0.15 g/l) or, where stated, in YE4S (yeast extract supplemented with adenine, leucine, histidine and uracil to a final concentration of 0.25 g/l) (Moreno et al., 1991). To shut off expression of the thiamine-repressible nmt41 promoter, thiamine hydrochloride was dissolved in water and then added to 30 mM. Cells were maintained in exponential growth for all experiments. Cells were grown at 25°C unless stated otherwise. Cell cycle arrests in G2 using *cdc2(as)* were performed with the addition of 1 μM 1-NmPP1 (Toronto Research Chemicals) for one cell cycle unless stated otherwise. Strains used in this work were authenticated by PCR and sequencing, and their full genotypes are listed in Table S1.

### Serial dilution assays

Cells were taken from a culture of exponentially growing cells at a density of 5×10^6^ cells/ml, which corresponds to the leftmost dilution of all dilution assays, followed by repeated 1:10 dilutions plated from left to right. For each spot, 4 μl of cell suspension was plated on EMM4S or YE4S. Plates were incubated for 3 days at 32°C unless otherwise stated.

### Fluorescence and time-lapse microscopy

Most imaging was performed using a Deltavision Elite (Applied Precision) microscope – an Olympus IX71 widefield inverted fluorescence microscope with a PLAN APO 60× oil, 1.42 NA objective and a Photometrics CoolSNAP HQ2 camera. The microscope was controlled using the SoftWoRx software. Widefield imaging of synCut3–mCherry strains was conducted using a Nikon Ti2 inverted microscope with Perfect Focus System and Okolab environmental chamber, and a Prime sCMOS camera (Photometrics). The microscope was controlled using Micro-Manager v2.0 software (Open Imaging). Fluorescence excitation was performed using a SpectraX LED light engine (Lumencor) fitted with a 575/25 filter for imaging mCherry. To maintain specified temperatures during imaging, an IMSOL incubator environment control system and an objective heater were used.

Slides for live-cell steady-state imaging were prepared by pelleting 1 ml of a >6×10^6^ cells/ml culture. The pellet was then resuspended in 3.5 μl of medium before 1.5 μl of this suspension was applied to a glass slide and covered with a coverslip. Fields of view (FOV) were avoided if they contained dead cells due to compression by the coverslip, but were otherwise sampled at random. For each slide, 10–20 FOV were acquired. FOV were imaged for <15 min to avoid imaging any perturbations resulting from slide-based acquisition. Imaging was optimised for signal intensity, as each FOV was only used once, and thus photobleaching and photo-toxicity were not of concern. Where Cut3–GFP or Cut3–tdTomato was used, CDK activity was assayed using the mean of the top 15% of pixel intensities within a cell body as an approximation of nuclear concentration. For images where Polo kinase activation was studied, the top eight pixel intensities within a cell body were used as a measure. Cell cycle stage assignment during widefield imaging was based on the nuclear:cytoplasmic (N:C) ratio of Cut3 by comparing the highest 15% of pixel intensities within a cell body with the lowest 85%, as an approximation of nuclear:cytoplasmic concentration. Cells possessing an N:C ratio of <0.95 were categorised as G2 cells. Cells possessing N:C ratios of 0.95–1.05 were categorised as G2-M cells. Where synCut3–mCherry was used, CDK activity was assayed by N:C ratio of the top 15% to bottom 85% of pixel intensities. Fiji was used for all image analysis (Schindelin et al., 2012).

MatTek glass-bottom dishes were used for time-lapse imaging applications. Dishes were pre-treated with soybean lectin to permit cell adherence. Before addition of cells, MatTek dishes were pre-warmed on a heat block at 32°C. Cells were grown and blocked in liquid culture before 2 ml of the culture was pelleted (2100 ***g*** for 30 s). Cell pellets were then resuspended in 1 ml of release medium (this point is defined as the time of release) in a new microcentrifuge tube before pelleting (2100 ***g*** for 30 s) and resuspension in 5 μl of medium. This concentrated cell suspension was then applied to the centre of the MatTek dish and allowed to settle for ∼5 s. The dish was then washed forcefully with 1 ml of release medium three times. The dish was then filled with 3 ml of release medium before rapid imaging. In general, the wash process required 1.5 min, and the imaging setup required 5 min for acquisition of ∼8 FOV.

## Supplementary Material

Supplementary information

Reviewer comments
