# Supplementary material for: A CDK activity buffer ensures mitotic completion
Source: J Cell Sci. 2022 Jun 21;135(12):jcs259626. doi: 10.1242/jcs.259626 (PMC9270952; doi:10.1242/jcs.259626)
Supplement: Supplementary information [file joces-135-259626-s1.pdf]

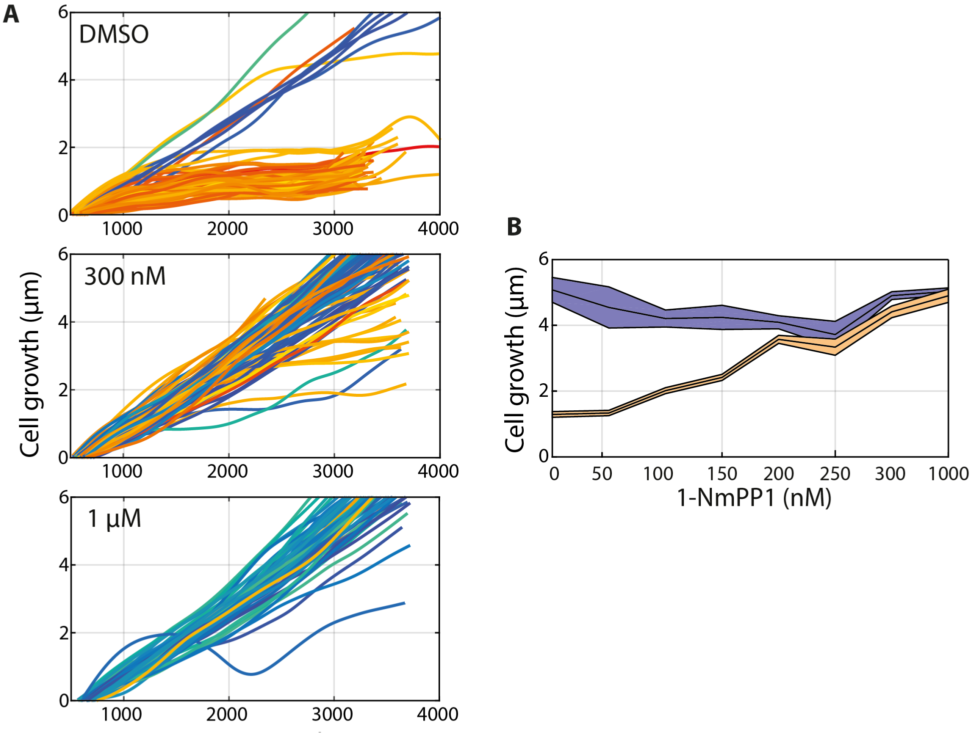

Fig. S1. CDK inhibition leads to a longer time before cell growth shut-off

A Cell growth after release from a 1µM 1-NmPP1 induced G2 block into the given concentrations of 1-NmPP1. Orange traces give cells that were automatically assigned as having entered mitosis based on their CDK activity profile (data derived from Figure 1D).

B Average cell growth after releasing from a 1µM 1-NmPP1 induced G2 block into annotated concentrations of 1-NmPP1. Length extension of cells that enter mitosis are given in orange, and length extension of cells that do not enter mitosis is given in blue. Shaded region indicated the standard error of the mean.

Journal of Cell Science • Supplementary information

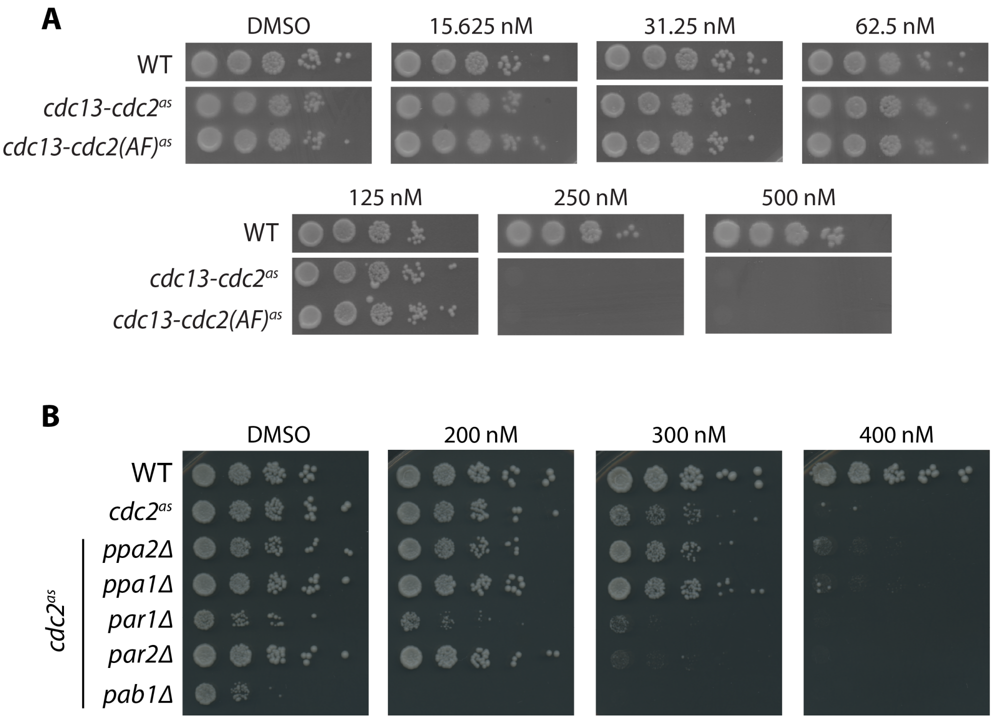

Fig. S2. Influence of CDK inhibitory phosphorylation and PP2A components on the CDK activity buffer

A Serial dilution assays of WT cells, and cells carrying Cdc13-Cdc2^{as} and Cdc13-Cdc2(AF)^{as} as their only source of CDK activity (in the absence of endogenous cyclins Cig1, Cig2, Puc1, Cdc13; and in the absence of endogenous Cdc2). Concentrations given above panels refer to 1-NmPP1 concentration. Cells were grown on EMM4S agar for 4 days at 32 °C.

B Serial dilution assays of WT cells, *cdc2*^{as} cells, and *cdc2*^{as} cells carrying deletions of the *ppa2*, *ppa1*, *par1*, *par2* and *pab1* gene. Concentrations given above panels refer to 1-NmPP1 concentration. Cells were grown on YE4S agar for 4 days at 25 °C.

Table S1. Strain list

Provides all strains used in this study with detailed genotypes, and source information.

Click here to download Table S1

Journal of Cell Science • Supplementary information
