# Supplementary material for: A CDK activity buffer ensures mitotic completion
Source: J Cell Sci. 2022 Jun 21;135(12):jcs259626. doi: 10.1242/jcs.259626 (PMC9270952; doi:10.1242/jcs.259626)
Supplement: Reviewer comments [file joces-135-259626_review_history.pdf]

Journal of Cell Science

A CDK activity buffer ensures mitotic completion

Souradeep Basu, James O. Patterson, Theresa U. Zeisner and Paul Nurse
DOI: 10.1242/jcs.259626

Editor: David Glover

Review timeline
Original submission: 23 November 2021
Editorial decision: 29 December 2021
First revision received: 12 April 2022
Accepted: 20 April 2022

Original submission

First decision letter

MS ID#: JOCES/2021/259626

MS TITLE: A CDK activity buffer ensures mitotic completion

AUTHORS: Souradeep Basu, James O Patterson, Theresa U Zeisner, and Paul Nurse
ARTICLE TYPE: Short Report

We have now reached a decision on the above manuscript.

To see the reviewers' reports and a copy of this decision letter, please go to: https://submit-jcs.biologists.org and click on the 'Manuscripts with Decisions' queue in the Author Area. (Corresponding author only has access to reviews.)

As you will see, the reviewers gave favourable reports but raised some critical points that will require amendments to your manuscript. I hope that you will be able to carry these out because I would like to be able to accept your paper, depending on further comments from reviewers.

We are aware that you may be experiencing disruption to the normal running of your lab that makes experimental revisions challenging. If it would be helpful, we encourage you to contact us to discuss your revision in greater detail. Please send us a point-by-point response indicating where you are able to address concerns raised (either experimentally or by changes to the text) and where you will not be able to do so within the normal timeframe of a revision. We will then provide further guidance. Please also note that we are happy to extend revision timeframes as necessary.

Please ensure that you clearly highlight all changes made in the revised manuscript. Please avoid using 'Tracked changes' in Word files as these are lost in PDF conversion.

I should be grateful if you would also provide a point-by-point response detailing how you have dealt with the points raised by the reviewers in the 'Response to Reviewers' box. Please attend to all of the reviewers' comments. If you do not agree with any of their criticisms or suggestions please explain clearly why this is so.

© 2022. Published by The Company of Biologists under the terms of the Creative Commons Attribution License (https://creativecommons.org/licenses/by/4.0/).

Reviewer 1

Advance summary and potential significance to field

This work by Basu et al. highlights the existence of a CDK activity buffer that allows ordered completion of mitosis even in conditions where CDK activity has been tampered with. Using an analog-sensitive allele of the only CDK in S. pombe (Cdc2), together with a marker of CDK activity (the ratio between nuclear and cytoplasmic Cut3), the authors show that cells can undergo mitosis in the presence of sublethal concentrations of the ATP-analog 1-NmPP1. The authors conclude that the maximum attainable CDK activity exceeds the activity required to complete mitosis, and this surplus renders the cells resistant to lower dosages of the inhibitor. Physiologically, the presence of this buffer could facilitate the division of cells grown in suboptimal conditions and that need to divide at a smaller cell size.
The authors also test the involvement of phosphatase activity in the buffer activity range. Surprisingly, they find that PP2A does not affect the degree of analog sensitivity, while deletion of clp1 (the S. pombe Cdc14-like phosphatase) increases the range.
Collectively, the results are compelling and interesting and I only have minor comments that could be corrected or further discussed in the text.

Comments for the author

The data presented and the conclusions derived from this work are convincing and contribute to a better understanding of mitotic control. I have only minor points to be addressed by the authors:
- In reference to Fig. 2E, the authors indicate that SPB separation occurs at similar levels of CDK activity in drug-treated cells. However, perhaps they should also mention that this is achieved at lower CDK activity in 1-NmPP1-treated cells than in the DMSO control.
- In Page 6, line 121, the authors refer to Aoi et al. for the metaphase arrest upon depletion of slp1 by means of a thiamine repressible promoter. As I recall it, in this paper they use a slp1 shut off system in meiosis in which they exchange the slp1 promoter with the rad21 promoter (encoding the mitotic cohesin kleisin subunit). I think the nmt41 system was first used by Petrova et al. (Quantitative analysis of chromosome condensation in fission yeast MCB 2013).
- In Fig. 3D: the legend for “time after release” is missing.
- From the plots in figure 3D and 3E it seems that the extra CDK activity attained in the metaphase arrest is more sensitive to the drug than CDK activity during a normal metaphase-anaphase transition. Could the authors comment on that?
- In lines 140-142, referring to Fig. 3D the authors state “Therefore, it appears that the maximum CDK activity observed in a cell cycle rises above that needed for mitotic completion”. Should it not be “maximum attainable”? The maximal activity that is achieved in the “Cdc20 ON” release is similar in mock-treated cells and in drug-treated cell up to 125 nM (and not higher). In normal conditions, CDK activity increases until reaching a certain threshold before entering mitosis, as shown in Fig. 1. If mitosis is prevented, as the authors point out, CDK activity can increase even further, showing the maximum CDK activity that can potentially be attained.
- In lines 214-216, the authors indicate that “deletion of PP2A, commonly thought as the major CDK-opposing phosphatase (Mochida et al., 2009), had little to no effect on the CDK activity buffer”. Since only 1-NmPP1 sensitivity was tested but no further experiments were done, perhaps this statement could be toned down.
- The authors do not elaborate much on the molecular mechanisms behind the CDK activity buffer. They show that it does not depend on Cdc2-Tyr15 phosphorylation, but how does it relate to Cdc13 accumulation?
The level of CDK activity does not reach a higher level in the control cells in Fig. 3 and only slightly higher in figure 1 (where no prior arrest has been imposed). However, in the latter figure, the slope of the curve before reaching peak activity differs between mock- and drug-treated cells. In my view, this could reflect the time required for cells to acquire sufficient Cdc13:Cdc2 complex while titrating the inhibitor. In figure 3, the pre-arrest in G2 would ensure that there is sufficient (albeit initially inactive) Cdc13:Cdc2 complex at time 0 h to buffer/titrate the inhibitor.
- The authors could comment on how the activity buffer is affected by cell size.

© 2022. Published by The Company of Biologists under the terms of the Creative Commons Attribution License (https://creativecommons.org/licenses/by/4.0/).

Reviewer 2

Advance summary and potential significance to field

In this manuscript Basu et al. report that during mitosis there is an excess of Cdk1/CyclinB activity ("a CDK activity buffer") that is used to ensure completion of the mitotic events. This is a new and interesting finding.

Comments for the author

In this manuscript Basu et al. report that during mitosis there is an excess of Cdk1/CyclinB activity ("a CDK activity buffer") that is used to ensure completion of the mitotic events. This is an interesting finding, most experiments in this manuscript are well executed and the data is very clear. However, I have some doubts about the interpretation of some experiments that need to be clarified before acceptance for publication in JCS.

Major point:
The experiments shown in figures 1D, 2 and 3 have been performed after arresting cdc2-as cells for 3 hours at the G2/M transition by adding 1 μM 1NM-PP1. During the arrest, cells continue to grow and accumulate inactive Cdc2as/Cdc13 complexes that upon release could generate high levels of CDK activity. This is known to be the case for instance during the arrest-release of the cdc25-22 mutant. Obviously, this is not a physiological situation and it is possible that the CDK activity buffer could be generated as a consequence of the G2 cell cycle arrest. Therefore, the authors should provide evidence that the CDK activity buffer is generated in an unperturbed cell cycle. Perhaps, they could film asynchronously growing wild type cells progressing from G2 into mitosis.

Other points:
1. The cdc2-as mutant used in this paper seems to be the originally described by Aoi et al. 2014. Open Biol. 4: 140063 containing two mutations, F84G and K79E. If this is the case, the authors should refer to this strain as cdc2-asM17 to avoid confusion with other cdc2-as strains constructed by other groups.
2. Another point that the authors could take into account when discussing their results is that phosphorylation of different mitotic CDK substrates could occur at different levels of CDK activity. Cut3 could be phosphorylated early with relatively low levels of CDK activity, whereas other mitotic CDK substrates could be phosphorylated late with higher CDK activity.
3. In relation to the phosphatases that counteract CDK activity in fission yeast, Cdc14-type (Clp1) and/or PP2A. The authors argue that Clp1 plays a role in regulating the CDK activity buffer whereas PP2A does not.
To conclude this, they use clp1Δ (lacking the Cdc14-type protein phosphatase activity) and ppa2Δ (lacking one of the two catalytic subunits of PP2A) mutants. In the second case (ppa2Δ), Ppa1 is still present and could contribute with sufficient PP2A activity to counteract the CDK buffer activity. On the other hand, it could be more interesting to discriminate between PP2A/B55 and PP2A/B56 activities by using mutants lacking the regulatory subunits of PP2A (Pab1, Par1 or Par2).
4. Supplementary Table S2 is missing in the website. Table S1 is not cited in the text.

Minor comments:
Page 3, line 69: "1 μm" should be "1 μM".
Page 5, line 101: "SPB (an approach that has been validated previously (Basu et al…" perhaps it is better
"SPB, an approach that has been validated previously by Basu et al…"
Page 9, line 205: "utilise use" should be "utilise" or "use".

© 2022. Published by The Company of Biologists under the terms of the Creative Commons Attribution License (https://creativecommons.org/licenses/by/4.0/).

First revision

Author response to reviewers' comments

We thank the reviewers and editor for their comments and suggestions on our manuscript. As a result, we have made changes to the text of our manuscript, and conducted additional experiments. We believe that our submission is significantly improved as a result of these comments and changes. We outline a point-by- point response below.

Reviewer 1

Advance Summary and Potential Significance to Field:

This work by Basu et al. highlights the existence of a CDK activity buffer that allows ordered completion of mitosis even in conditions where CDK activity has been tampered with. Using an analog-sensitive allele of the only CDK in S. pombe (Cdc2), together with a marker of CDK activity (the ratio between nuclear and cytoplasmic Cut3), the authors show that cells can undergo mitosis in the presence of sublethal concentrations of the ATP-analog 1-NmPP1.

The authors conclude that the maximum attainable CDK activity exceeds the activity required to complete mitosis, and this surplus renders the cells resistant to lower dosages of the inhibitor. Physiologically, the presence of this buffer could facilitate the division of cells grown in suboptimal conditions and that need to divide at a smaller cell size. The authors also test the involvement of phosphatase activity in the buffer activity range. Surprisingly, they find that PP2A does not affect the degree of analog sensitivity, while deletion of clp1 (the S. pombe Cdc14-like phosphatase) increases the range. Collectively, the results are compelling and interesting and I only have minor comments that could be corrected or further discussed in the text.

Reviewer 1 Comments for the Author:

The data presented and the conclusions derived from this work are convincing and contribute to a better understanding of mitotic control. I have only minor points to be addressed by the authors:

-In reference to Fig. 2E, the authors indicate that SPB separation occurs at similar levels of CDK activity in drug- treated cells. However, perhaps they should also mention that this is achieved at lower CDK activity in 1-NmPP1- treated cells than in the DMSO control.

We have now clarified that 1-NmPP1 treated cells achieve SPB separation at a lower, but consistent, CDK activity level than the DMSO control [line 92-94].

-In Page 6, line 121, the authors refer to Aoi et al. for the metaphase arrest upon depletion of slp1 by means of a thiamine repressible promoter. As I recall it, in this paper they use a slp1 shut off system in meiosis in which they exchange the slp1 promoter with the rad21 promoter (encoding the mitotic cohesin kleisin subunit). I think the nmt41 system was first used by Petrova et al. (Quantitative analysis of chromosome condensation in fission yeast, MCB 2013).

The reviewer is correct, and we have changed the reference from Aoi *et al*. to Petrova *et al*. accordingly [line 108].

-In Fig. 3D: the legend for “time after release” is missing.

Fixed and added.

-From the plots in figure 3D and 3E it seems that the extra CDK activity attained in the metaphase arrest is more sensitive to the drug than CDK activity during a normal metaphase-anaphase transition. Could the authors comment on that?

© 2022. Published by The Company of Biologists under the terms of the Creative Commons Attribution License (https://creativecommons.org/licenses/by/4.0/).

We believe the insensitivity of CDK activity to CDK inhibition in the Slp1-ON condition (no metaphase arrest) to be due to the CDK activity buffer. In this scenario, the activity buffer allows a normal mitotic progression until the buffer is depleted. We now outline this view fully on lines 128-133.

-In lines 140-142, referring to Fig. 3D the authors state "Therefore, it appears that the maximum CDK activity observed in a cell cycle rises above that needed for mitotic completion". Should it not be "maximum attainable"? The maximal activity that is achieved in the "Cdc20 ON" release is similar in mock-treated cells and in drug-treated cell up to 125 nM (and not higher). In normal conditions, CDK activity increases until reaching a certain threshold before entering mitosis, as shown in Fig. 1. If mitosis is prevented, as the authors point out, CDK activity can increase even further, showing the maximum CDK activity that can potentially be attained.

The reviewer is correct, and we have now corrected this on line 127-128.

-In lines 214-216, the authors indicate that "deletion of PP2A, commonly thought as the major CDK-opposing phosphatase (Mochida et al., 2009), had little to no effect on the CDK activity buffer". Since only 1-NmPP1 sensitivity was tested but no further experiments were done, perhaps this statement could be toned down.

Reviewer 2 also raised points about the deletion of PP2A, and we have resolved this issue later in our response. In brief, Reviewer 1 was correct to raise this point as, when we conducted further experiments with finer 1-NmPP1 concentrations, we indeed found an effect of PP2A deletion and have adjusted our conclusions accordingly.

-The authors do not elaborate much on the molecular mechanisms behind the CDK activity buffer. They show that it does not depend on Cdc2-Tyr15 phosphorylation, but how does it relate to Cdc13 accumulation? The level of CDK activity does not reach a higher level in the control cells in Fig. 3 and only slightly higher in figure 1 (where no prior arrest has been imposed). However, in the latter figure, the slope of the curve before reaching peak activity differs between mock- and drug-treated cells. In my view, this could reflect the time required for cells to acquire sufficient Cdc13:Cdc2 complex while titrating the inhibitor. In figure 3, the pre-arrest in G2 would ensure that there is sufficient (albeit initially inactive) Cdc13:Cdc2 complex at time 0 h to buffer/titrate the inhibitor.

The reviewer raises an important consideration, and one that we are trying to understand currently. However, fully addressing this requires interfering with Cdc13 accumulation, which is beyond the scope of this work.

Given that discussion of molecular mechanisms would lead to a confusing and incomplete argument, we have opted not to delve into these issues in the discussion. Instead, we simply outline that we do not completely understand the molecular mechanism of the buffer, but that it may relate to Cdc13:Cdc2 accumulation, or other factors that influence CDK activity [lines 200-207].

-The authors could comment on how the activity buffer is affected by cell size.

We have included how cell size may influence the buffer region with our changes outlined above.

Reviewer 2 Advance Summary and Potential Significance to Field:

In this manuscript Basu et al. report that during mitosis there is an excess of Cdk1/CyclinB activity ("a CDK activity buffer") that is used to ensure completion of the mitotic events. This is a new and interesting finding.

© 2022. Published by The Company of Biologists under the terms of the Creative Commons Attribution License (https://creativecommons.org/licenses/by/4.0/).

Reviewer 2 Comments for the Author:

In this manuscript Basu et al. report that during mitosis there is an excess of Cdk1/CyclinB activity ("a CDK activity buffer") that is used to ensure completion of the mitotic events. This is an interesting finding, most experiments in this manuscript are well executed and the data is very clear. However, I have some doubts about the interpretation of some experiments that need to be clarified before acceptance for publication in JCS.

Major point:
The experiments shown in figures 1D, 2 and 3 have been performed after arresting cdc2-as cells for 3 hours at the G2/M transition by adding 1 μM 1NM-PP1. During the arrest, cells continue to grow and accumulate inactive Cdc2as/Cdc13 complexes that upon release could generate high levels of CDK activity. This is known to be the case for instance during the arrest-release of the cdc25-22 mutant. Obviously, this is not a physiological situation and it is possible that the CDK activity buffer could be generated as a consequence of the G2 cell cycle arrest. Therefore, the authors should provide evidence that the CDK activity buffer is generated in an unperturbed cell cycle. Perhaps, they could film asynchronously growing wild type cells progressing from G2 into mitosis.

While the reviewer is correct that in the experiments highlighted we use arrested cells, in Figure 1 B&C we use unperturbed cells and show that the CDK activity buffer is present as cells remain viable in low concentrations of 1-NmPP1. This suggests that maximal attainable CDK activity in unperturbed cells that have not been G2 arrested is likely to exceed the level needed for mitosis. We feel that this provides evidence that the CDK activity buffer is generated in the absence of a G2 arrest.

In addition, the concentration of 1-NmPP1 that leads to a loss of cell viability in unperturbed cells in Figure 1B corresponds well to the concentration at which G2 arrested cells become unable to reach the mitotic CDK activity threshold in Figure 3 (~250 nM 1-NmPP1). This suggests that the extended G2 block is unlikely to be the source of the CDK activity buffer, as the ability of the buffer region to withstand CDK inhibition is similar between blocked and unperturbed cells.

We agree with the reviewer that accumulation of cyclin-CDK may underlie the molecular mechanism of the buffer, or perhaps be related to other factors related to cell size or CDK activity, which is a point we have now incorporated into our discussion [line 204-207].

Other points:
1. The cdc2-as mutant used in this paper seems to be the originally described by Aoi et al. 2014. Open Biol.4: 140063 containing two mutations, F84G and K79E. If this is the case, the authors should refer to this strain as cdc2-asM17 to avoid confusion with other cdc2-as strains constructed by other groups.

The reviewer is correct, and we now clarify that the *cdc2* allele used is indeed the *cdc2-asM17* allele at first usage, but prefer to use *cdc2(as)* throughout otherwise for ease of understanding to non-specialists [line 59].

2. Another point that the authors could take into account when discussing their results is that phosphorylation of different mitotic CDK substrates could occur at different levels of CDK activity. Cut3 could be phosphorylated early with relatively low levels of CDK activity, whereas other mitotic CDK substrates could be phosphorylated late with higher CDK activity.

The reviewer raises an important consideration when interpreting our Cut3 results. We have now added lines to our discussion reflecting this limitation, as it is known that CDK substrate sensitivity to CDK activity varies by orders of magnitude. Given we are particularly interested in the phosphorylation of the final essential mitotic substrate, we state that Cut3 phosphorylation is unlikely to represent this [lines 186-191].

3. In relation to the phosphatases that counteract CDK activity in fission yeast, Cdc14-type (Clp1) and/or PP2A. The authors argue that Clp1 plays a role in regulating the CDK activity

© 2022. Published by The Company of Biologists under the terms of the Creative Commons Attribution License (https://creativecommons.org/licenses/by/4.0/).

buffer whereas PP2A does not. To conclude this, they use clp1Δ (lacking the Cdc14-type protein phosphatase activity) and ppa2Δ (lacking one of the two catalytic subunits of PP2A) mutants. In the second case (ppa2Δ), Ppa1 is still present and could contribute with sufficient PP2A activity to counteract the CDK buffer activity. On the other hand, it could be more interesting to discriminate between PP2A/B55 and PP2A/B56 activities by using mutants lacking the regulatory subunits of PP2A (Pab1, Par1 or Par2).

Considering the literature on PP2A we were surprised when the *ppa2Δ* did not have an effect on viability at 400 nM, while the *clp1Δ* rescued the lethality of 400nM 1-NmPP1 treatment. We initially chose 400 nM 1-NmPP1 as it was the lowest concentration where *cdc2(as)* cells definitively lost viability.

Following the reviewer's comments, we examined an intermediate concentration, 300 nM, where deletion of the PP2A subunit *ppa2* led to an increase in viability, suggesting that the PP2A subunit encoded by *ppa2* does have an impact on extending the buffer, but to a lesser extent than removal of Clp1 (Figure R1). We have now rephrased our initial observations [lines 155-157] and discussion [lines 210-211] to reflect that *ppa2* deletion does have an effect on the activity buffer, and added Figure R1 as Figure 4A.

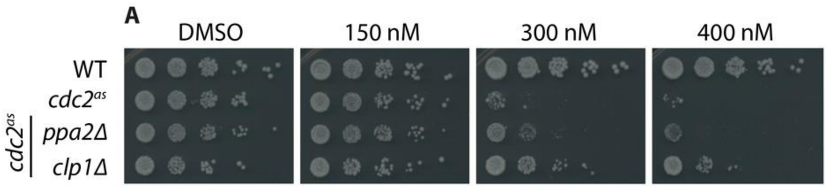

Figure R1: Serial dilution assay of WT cells, *cdc2*^{as} cells, and *cdc2*^{as} cells carrying deletions of the *ppa2* gene (*ppa2Δ*), and the *clp1* gene (*clp1Δ*). Concentrations given above panels refer to 1-NmPP1 concentration. Cells were grown on YE4S agar for 4 days at 25 ^{O}C.

We also tested the deletion of *ppa1*, *pab1*, *par1*, and *par2* to see if deletion of the alternative catalytic subunit or the B55/B56 targeting subunits had the ability to extend the CDK activity buffer. Although deletion of the targeting subunits did not result in increased viability in 1-NmPP1, deletion of *ppa1* had a similar effect to the deletion of *ppa2* (Figure R2). However, we were unable to test the *ppa1Δ ppa2Δ* double deletion strain as it is inviable. We have now included these results as Figure S2B, and explain them in the text on lines 160-168. In the discussion, we also now also state that although removal of Clp1 has the greatest effect on the CDK activity buffer on its own, compensation between PP2A catalytic subunits may mask the true effect of PP2A activity on the CDK activity buffer [lines 212-213].

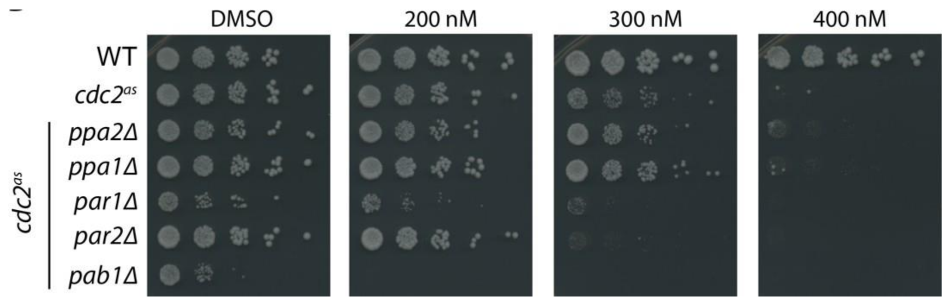

Figure R2: Serial dilution assays of WT cells, *cdc2*^{as} cells, and *cdc2*^{as} cells carrying deletions of the *ppa2*, *ppa1*, *par1*, *par2* and *pab1* gene. Concentrations given above panels refer to 1-NmPP1 concentration. Cells were grown on YE4S agar for 4 days at 25 ^{O}C.

4. Supplementary Table S2 is missing in the website. Table S1 is not cited in the text.

© 2022. Published by The Company of Biologists under the terms of the Creative Commons Attribution License (https://creativecommons.org/licenses/by/4.0/).

We apologise for this typo. It should read Table S1 and has now been fixed. There should only be one supplementary table.

Minor comments:
Page 3, line 69: “1 µm” should be “1 µM”.

Corrected [line 63]

Page 5, line 101: “SPB (an approach that has been validated previously (Basu et al…” perhaps it is better “SPB, an approach that has been validated previously by Basu et al…”

We have now moved these references to the end of the line [line 92]

Page 9, line 205: “utilise use” should be “utilise” or “use”.

Corrected [line 199]

Second decision letter

MS ID#: JOCES/2021/259626

MS TITLE: A CDK activity buffer ensures mitotic completion

AUTHORS: Souradeep Basu, James O Patterson, Theresa U Zeisner, and Paul Nurse
ARTICLE TYPE: Short Report

I am happy to tell you that your manuscript has been accepted for publication in Journal of Cell Science, pending standard ethics checks.

© 2022. Published by The Company of Biologists under the terms of the Creative Commons Attribution License (https://creativecommons.org/licenses/by/4.0/).
